# Effects of Aged Oil Sludge on Soil Physicochemical Properties and Fungal Diversity Revealed by High-Throughput Sequencing Analysis

**DOI:** 10.1155/2018/9264259

**Published:** 2018-09-06

**Authors:** Huihui Wang, Shaoping Kuang, Qiaolin Lang, Wenjuan Yu

**Affiliations:** ^1^College of Environment and Safety Engineering, Qingdao University of Science and Technology, Qingdao, 266042 Shandong Province, China; ^2^Shandong Provincial Key Laboratory of Eco-Environmental Science for Yellow River Delta, Binzhou University, Binzhou, 256600 Shandong Province, China; ^3^College of Chemistry and Molecular Engineering, Qingdao University of Science and Technology, Qingdao, 266042 Shandong Province, China

## Abstract

The oilfield soil was contaminated for years by large quantities of aged oil sludge generated in the petroleum industry. In this study, physicochemical properties, contents of main pollutants, and fungal diversity of the aged oil sludge-contaminated soil were analyzed. Results revealed that aged oil sludge significantly changed physical and chemical properties of the receiving soil and increased the contents of main pollutants (petroleum hydrocarbons and heavy metals) in soil. Meanwhile, the internal transcribed spacer (ITS) sequencing by Illumina Miseq platform at each taxonomic level demonstrated that the toxicological effect of oil pollutants obviously influenced the fungal diversity and community structure in soil. Moreover, it was found that the presence of three genera (*Cephalotheca*, *Lecanicillium*, and *Septoriella*) appeared in aged oil sludge-contaminated soil. And oil pollutants promoted the growth of certain genera in *Ascomycota* (70.83%) and *Basidiomycota* (10.78%), such as *Venturia*, *Alternaria*, and *Piloderma*. Nevertheless, the growth of *Mortierella* (9.16%), *Emericella* (6.02%), and *Bjerkandera* (0.00%) was intensively limited. This study would aid thorough understanding of microbial diversity in oil-contaminated soil and thus provide new point of view to soil bioremediation.

## 1. Introduction

Oil sludge is one of the most significant hazardous solid wastes generated in oil industry in China [1]. It was mainly generated from the process of drilling, exploitation, transportation, refining, and storage of crude oil. A large amount of oil sludge consisted of many hazardous chemicals, such as petroleum hydrocarbons (PHCs) and heavy metals (HMs), which are of great concern for the potential toxicity to human, have been emitted into the local environment [2–5]. On the receiving soils, the toxic components in oil sludge may cause nutrient deficiency or limit the growth of seed and plants [6]. Oil sludge was stacked in the open air with continually decreasing of volatile components (mainly some light oil components) and moisture content, generating quantities of aged oil sludge (AOS) [7]. As time passed, the hazardous chemicals in AOS were continuously emitted into the local environment, resulting in chronic pollution effects on the receiving soil. Compared with oil sludge, AOS has the characteristics of higher heavy oil content, longer pollution period, and less comprehensive utilization [7].

In previous studies, much attention has been paid to the proper disposal and sufficient treatment of the stacking oil sludge [7, 8]. The spilled oil sludge in oil-producing regions, especially around the oil wells, was ignored for years or even decades, forming lots of AOS-contaminated sites on soil. Until now, as the most deleterious components in AOS [9], the contents of total petroleum hydrocarbons (TPHs) and HMs as well as fungal diversity of the long-time oil-polluted soil are still unknown and rarely referred and reported globally.

In recent decades, within the various biological techniques, high-throughput sequencing technology with better capacity for detecting rare species [10] is identified as a highly efficient tool for researching the entire profile of microorganism community [11]. It has been proved that soil microorganisms are far more sensitive to pollution than soil, animals, or plants [12–14], with the evidence that the microorganisms in oil-contaminated soil are obviously different from those in background soil [15, 16]. Some domain bacteria phyla like *proteobacteria* are found in different contaminated receptors, such as soil [17] and activated sludge [11]. However, as a good indicator of pollution, fungal diversity in contaminated soil is rarely reported.

This is the first study that took fungi as the typical indicators of pollution to evaluate the microbial variations in soil caused by AOS. In this study, an AOS site of 4 years on soil was selected as the point source pollution of sampling, in which the fungal diversity and community structure in soil were explored with the method of high-throughput sequencing technique. This study is aimed at evaluating the influence from AOS on physicochemical properties, fungal community structure, and diversity in soil, as well as screening dominant or core oil-resistant fungal genera for potential use in soil bioremediation. The results and related findings would aid in thorough understanding of microorganism structure in oil-contaminated soil and provide new point of view to soil bioremediation.

## 2. Materials and Methods

### 2.1. Sampling Site

The samples of aged oil sludge were obtained from Gudao oil factory, the largest output plant of crude oil in Shengli oilfield. Gudao lies in semiarid warm temperate monsoon climate zone at latitudes 37°47′ N to 37°84′ N and longitudes 118°39′ E to 119°8′ E. The soil in Gudao is saline-alkali and raises reeds as the major vegetation. Gudao locates inside the region of Yellow River Delta, in which there is a National Nature Reserve with hundreds of protected animals and plants distributed over 4500 km^2^ of wetlands. For the reason of oil exploration, there are many oil wells in Gudao and lots of spilled oil sludge sites on soil, resulting in relatively small and decentralized AOS sites around oil wells.

### 2.2. Experimental Setup

**Figure 1:**
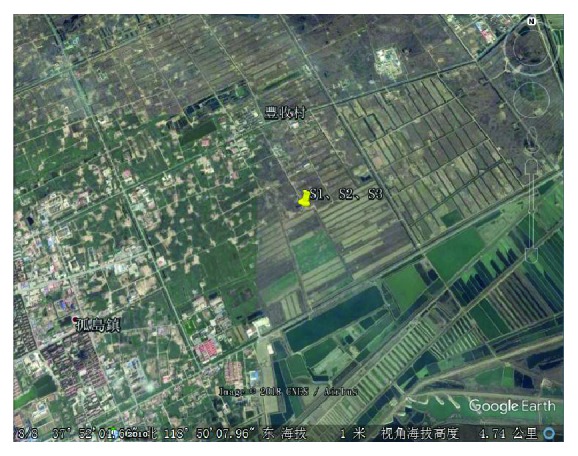
Sampling sites.

An AOS spot around an oil well drilled 4 years ago with the approximate diameter of 40 cm was selected. Two soil samples at the horizontal center of AOS spot were collected from 0 cm and 20 cm vertically below the surface of soil, respectively (labeled as S1 and S2). Then, another soil sample obtained from the surface soil and 120 m away from the AOS center without oily sludge surroundings was chosen as blank (labeled as S3, uncontaminated soil sample) (Figure 1). All the samples were stored in an ice chest with stones and plant residues removed, carried backed to the laboratory within 4 hours. Those soil samples were divided into two parts. One part was used for the determination of physicochemical properties, HMs and TPHs (air-dried and processed with a 100-mesh sieve). The other part was analyzed by high-throughput sequencing.

### 2.3. Analytical Methods

#### 2.3.1. Determination of Soil Physicochemical Properties

The pH was tested in deionized water at a soil/water solution ratio of 1 : 2.5 using a pH meter (Mettler-Toledo Instruments, Shanghai, China) [18]. Moisture content was determined with gravimetric method by weighing samples before and after oven-drying at 105°C for 24 h [19]. Salinity was determined by the difference in weight of the solid after the process of washing, filtration, oxidation with H_2_O_2_, and drying at 100~105°C to a constant weight. The Walkley-Black method was used in this study to determine the dry mass of organic carbons in soil samples [17].

Contents of heavy metals (copper, zinc, and chromium) were determined by atomic absorption spectrophotometer (AAS7000, SHIMADZU, Japan), with pretreatment of digesting by nitric acid, hydrofluoric acid, and hydrogen peroxide system (5 : 2 : 1 by volume), respectively, in microwave digestion instrument [20]. Contents of TPHs were measured using a Purge & Trap Sample Concentrator (Eclipse 4660, OI, USA) combined with GC (7890A with FID detector, Agilent Technologies) [21].

#### 2.3.2. Extraction of Genomic DNA and PCR Amplification

Genomic DNA of the three soil samples were isolated and extracted using the soil DNA kit (Omega Bio-tek, Norcross, GA, USA) following the manufacturer's protocols. The DNA extracts were stored at −20°C for the PCR amplification (95°C for 5 min, followed by 27 cycles at 95°C for 30 s, 55°C for 30 s, and 72°C for 45 s and a final extension at 72°C for 5 min) which was performed in an ABI GeneAmp 9700 (USA). The fungal rDNA-ITS region was amplified using universal primers ITS1F (5′-CTTGGTCATTTAGAGGAAGTAA-3′) and ITS2R (5′-GCTGCGTTCTTCATCGATGC-3′) where barcode is an eight-base sequence unique to each sample. PCR reactions were performed in triplicate 20 *μ*L mixture containing 4 *μ*L of 5x FastPfu buffer, 2 *μ*L of 2.5 mM dNTPs, 0.8 *μ*L of each primer (5 *μ*M), 0.4 *μ*L of FastPfu polymerase, and 10 ng of template DNA.

#### 2.3.3. Illumina Miseq PE2500 Sequencing

Amplicons were extracted from 2% agarose gels and purified using the AxyPrep DNA Gel Extraction Kit (Axygen Biosciences, Union City, CA, USA) according to the manufacturer's instructions and quantified using QuantiFluor: trademark: -ST (Promega, USA). Purified amplicons were pooled in equimolar and paired-end sequenced (2 × 250) on an Illumina Miseq platform according to the standard protocols.

#### 2.3.4. Data Analysis

Raw data must be processed to remove the low-quality data [22] for further analysis. After removing the adaptors, primers and low-quality reads, the pair-end reads were overlapped to assemble the final sequences using the FLASH software. The criterions of overlapping were that the overlapping lengths were >10 bp and the default threshold values were ≤0.2. Chimera tags were further filtered out using the Gold database by UCHIME (version 4.2.40), and finally the effective tags were generated. The operational taxonomic unit (OTU) analysis was performed using the Uparse package (version 7.0.1001) with a 97% sequence identity. Each OTU was taxonomically assigned to the UNITE database using the ribosomal database project (RDP) classifier. OTUs were processed by removing chloroplast sequences, chondriosome sequences, and unclassified sequences. Finally, the OTUs with relative abundance of above 1% were retained.

The Shannon-Weaver diversity index (*H*) and Simpson index (*D*) were used to express the diversity of the soil fungal community, which are calculated as follows:

(1) $$\begin{array}{c} H_{Shannon}=-\overset{S_{obs}}{\underset{i=1}{\sum }}\frac{n_{i}}{N}\ln\frac{n_{i}}{N},\\ D_{Simpson}=\frac{\sum_{i=1}^{S_{obs}}n_{i}\left(n_{i}-1\right)}{N\left(N-1\right)}, \end{array}$$

where *S*_obs_ is the number of OTUs, *n*_*i*_ is the number of sequence in OTU_*i*_, and *N* is the sum of all the sequences in OTUs.

All the determinations were performed at least in triplicate. Statistical significance was determined at the confidence levels of 0.05.

## 3. Results and Discussion

### 3.1. Overview of Changes in Soil Physicochemical Properties

As a result of its high viscosity, aged oil sludge can be fixed in soil pores or adsorbed onto the surface of soil mineral constituents, causing reduction of water retention capacity and hydraulic conductivity of the soil [1, 23, 24]. It would lead to ultimate change in physical and chemical properties of oil receiving soil. The main physicochemical properties of the samples were determined and listed in Table 1. It was observed that the pH values of the three soil samples ranged from 8.11 to 8.56. The salinity of S1 (0.27%) and S2 (0.36%) was much lower than that of S3 (1.45%) which indicated a higher biomass in the AOS-contaminated soil [15]. The total organic carbon (TOC) in S1 and S2 was much higher than that in S3 due to the oil input. The moisture contents in S1 (21.05%) and S2 (22.55%) were generally higher compared with S3 (13.95%) which was inconsistent with previous studies [1, 7]. It is probably because that hydrophobic crusts formed by the heavy oil components in AOS limited the evaporation of water and water/air exchange of soil [25]. Therefore, the soil around AOS discharged was arid and saline-alkali soil; meanwhile, its physical and chemical properties were significantly changed by AOS. It has been demonstrated that differences in edaphic properties, such as pH and moisture, are often associated with differences in soil fungal communities, not only in richness but also in composition and structure [17, 26, 27].

Along with the emission of oil sludge to the receiving soil, the contents of TPHs and HMs were intensively increased. The concentrations of Cu, Zn, and Cr in S1 were approximately the same with those in S2, while 6.3, 8.4, and 2.2 times of those in S3, respectively (Table 1). Most heavy metals had a cumulative effect and were of particular hazard to ecological receptors and humans [1]. The fungal communities were reported strongly sensitive to the presence of HMs [28] and have higher toleration to metal pollutants than bacteria [20]. On the other side, the contents of TPHs of S1 and S2 were 15.2 mg/kg and 13.6 mg/kg, respectively. While in S3, there was no (or under detection limit of method) TPHs detected. Higher concentrations of TPHs have been reported showing stronger toxic effects on the activity of soil enzymes and microorganisms [23]. In particular, the polycyclic aromatic hydrocarbons (PAHs) in AOS were of great concern for genotoxicity to human and could migrate to groundwater through soil profile [13, 29]. Furthermore, it was found that there was joint toxic effect between PAHs and HMs in AOS-contaminated soil, which was studied by our research group.

### 3.2. Miseq Sequencing Results and Fungal Community Structures

By amplifying the ITS region of fungi, Illumina high-throughput sequencing which adopted a sequencing-by-synthesis approach [30] enabled thorough identification of fungal community structures, including those that could not be cultured or detected in traditional approaches [31–34]. Totally, 96,331 valid sequences of the ITS gene were obtained with an average length of 241 bp. RDP classifier was used in hierarchical clustering analysis at the similarity threshold of 97%. The sequence information and fungal diversity indexes are listed in Table 2. The numbers of OTUs, Ace, Chao 1, the Shannon-Weaver indexes, and Simpson indexes of the three samples showed obvious change after AOS exposure. The numbers of OTUs, Ace, and Chao1 indexes reduced from S3 to S2 and S1, indicating less OTUs and species richness in contaminated soils [35]. S1 had the lowest Shannon-Weaver index (4.36) and the highest Simpson index (0.0467), suggested smallest fungal community diversity [35]. While the Shannon-Weaver index (4.61) and Simpson index (0.0355) of S2 reflected the biggest fungal community diversity. The Good's coverage estimator was used to assess the sampling completeness, obtaining the results of above 0.999, indicating an appropriate reveal of most fungal diversity in all samples [36, 37].

**Figure 2:**
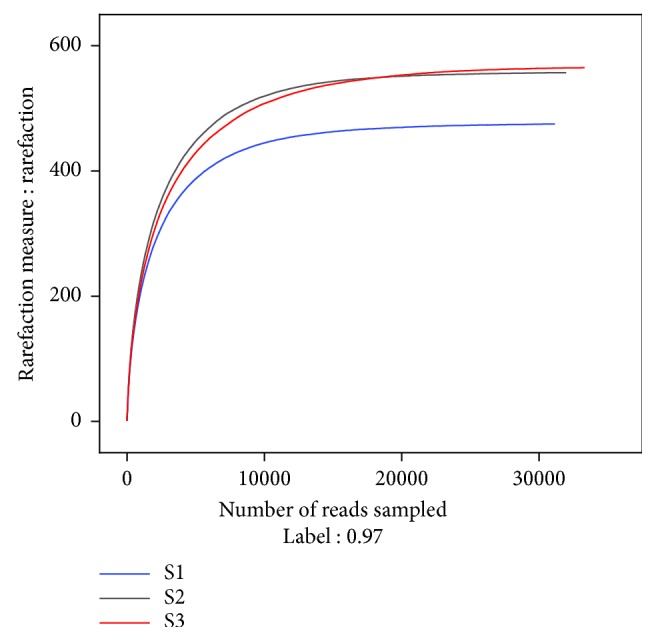
Rarefaction curves based on the 18s rRNA gene sequencing.

**Figure 3:**
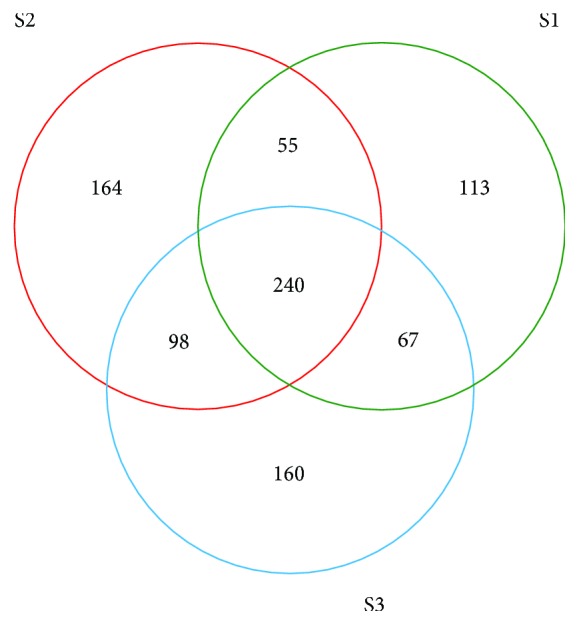
OTU venn analysis in different samples.

The rarefaction analysis was used to verify whether the volume or the depth of sampling was sufficient to capture the existing OTUs [22, 36]. As shown in Figure 2, the three soil samples generally had the same patterns of rarefaction curves that showed a trend to level off, indicating a sufficient sampling of the fungal species. The Venn diagram was used to analyze the species composition by evaluating the distribution of fungal community. It could provide direct expression of the similarity and overlapping numbers of OTUs between different samples. As shown in Figure 3, the numbers of OTUs in S1, S2, and S3 were 475, 557, and 565, separately, among which 240 OTUs accounted for 73.09% of the total sequences were shared, and 168 OUTs in S1 and 219 OTUs in S2 were unique, respectively, after AOS exposure. Meanwhile, comparing to S3, 160 OTUs disappeared in the contaminated soils. Therefore, AOS affected the richness of fungal community and changed fungal diversity to a certain extent.

### 3.3. Taxonomic Complexity of Fungal Community

**Figure 4:**
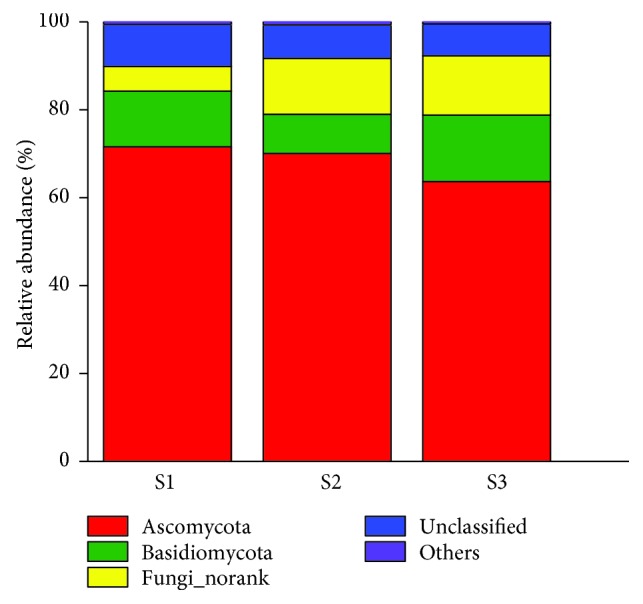
Histogram of fungal community structure at phylum level.

The fungal community compositions of the three soil samples reflected similar diversities but different abundances.  Figure 4 provided the fungal community information in phylum level. Among the total 6 identified phyla, *Ascomycota* was the most abundant phylum followed by *Basidiomycota* in all samples. *Ascomycota* has been reported as the largest phylum by far with over 64,000 identified species in almost 6400 genera [38] and the most typical dominant phylum in soil [39]. In this study, *Ascomycota* accounted for 63.67% of total DNA sequences in the noncontaminated soil (S3). While in the contaminated soil samples, the percentage of *Ascomycota* increased to 71.58% in S1 and 70.08% in S2, indicating that oily sludge was beneficial to the existence of *Ascomycota*. According to Aranda [40], PAH-polluted soils were found to be colonized mostly by *Ascomycota* and indigenous ascomycete was able to transform or remove PAHs. PAHs are the main components of the hydrocarbons in AOS. Therefore, it might explain the enrichment of *Ascomycota* in soils after long-term AOS exposure. The *Basidiomycota* has been recognized as prominent tool for the degradation of recalcitrant pollutants due to their rich supply of laccase, tyrosinase, and soluble extracellular enzymes, such as lignin-modifying enzyme (LME) [40, 41]. In this study, we observed a decrease of the percentage of *Basidiomycota* (by −2.46% in S1 and −6.26% in S2) in contaminated soils compared with S3 (15.13%). Although the growth was limited, *Basidiomycota* was still the second largest phyla in the contaminated soils.

Further analysis was carried out to analyze the fungal community composition in family levels. In general, 91.83% of the total sequences were assigned and there were 28 identified families with the relative abundance of above 1%. As shown in Table 3, S1 and S2 had similar family structures and diversities. In the noncontaminated soil (S3), the top three abundant families were Mortierellaceae (13.46%), Aspergi laceae (11.32%), and Meruliaceae (9.33%). While in contaminated soils, the growth of the above families was powerfully limited and their percentages in S1 reduced to 5.58%, 10.76%, and even 0.00%, respectively. In contrast, the proportion of certain families was significantly increased in contaminated soils compared with S3, such as Cephalothecaceae, Cordycipitaceae, Pleosporaceae, and Thelephoraceae. Especially the family of Cephalothecaceae, as the largest family among all the identified families, it was a new-appeared fungal family in contaminated soils with the abundance of 16.76% in S1 and 12.63% in S2. The family Cephalothecaceae in Sordariomycetes (Ascomycota) was incertae sedis [42] because of uncertain phylogenetic placement and differing morphology [43]. In addition, Cordycipitaceae and Pleosporaceae are members of Ascomycota, while Thelephoraceae belongs in Basidiomycota.

**Figure 5:**
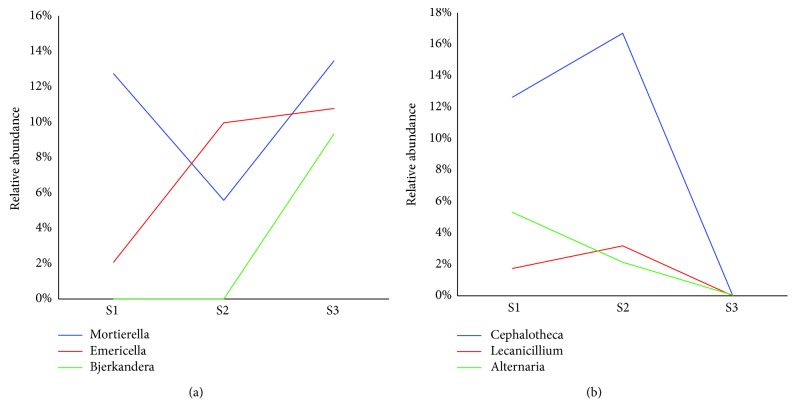
Relative abundance of the three (a) limited genera and (b) oil-resistant genera in samples.

The relative fungal abundance at genus level was also analyzed. Totally, 39 genera with the abundance of above 1% were classified in the samples. It was observed that the growth of most identified genera was limited, with the possible reason that AOS discharged TPHs into the receiving soil. TPHs comprised hydrogen and carbon, but lack of nitrogen, sulfur, and phosphorus essential for microbial growth [44]. As the dominant fungi in S3, the growth of *Mortierella* (13.46%), *Emericella* (10.77%), and *Bjerkandera* (9.33%) presented a decreased dynamic to 12.74%, 2.08%, and 0.00% in S1, respectively (Figure 5(a)), while *Alternaria*, *Cephalotheca*, and *Lecanicillium* with the abundances of 5.31%, 12.63%, and 1.74% in S1 was found oil-tolerant (the relative abundances in S3 were 0.05%, 0.00%, and 0.00%) (Figure 5(b)). In general, there were three new genera (*Cephalotheca*, *Lecanicillium*, and *Septoriella*) which did not exist in noncontaminated soil (0.00%), and another three genera (*Venturia*, *Alternaria*, and *Piloderma*) almost not found in S3 of which the percentage was below 0.1% of the total genera. *Cephalotheca* was the genus of fungi in the *Cephalothecaceae* family of the *Ascomycota*. It was the core genus with high abundance of 12.63% (in S1) and 16.70% (in S2) in AOS-contaminated soils. *Lecanicillium* is well known as entomopathogenic fungal species. Temperature between 10 and 25°C and higher moisture are benefit to conidial germination of *Lecanicillium* spp. [45]. Considering that the average temperature of Shengli oilfield was 12.9°C, and the AOS-contaminated soils had higher moisture contents than noncontaminated soil (Table 1), it might be the reason for the appearance of *Lecanicillium*. Furthermore, *Septoriella*, *Venturia*, and *Alternaria* were the genera of fungi in *Ascomycota*, and *Piloderma* was the genus of fungi in *Basidiomycota* that could adapt oily circumstance. They are the promising fungi genera that could be used in bioremediation of aged oil contamination in the future.

**Figure 6:**
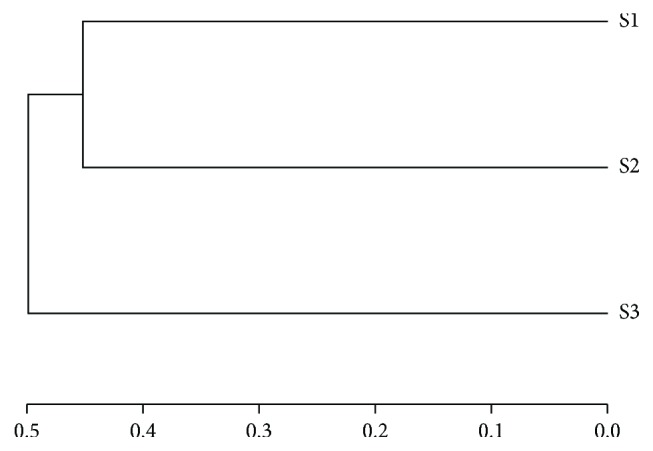
Multiple sample cluster tree.

The analysis of multiple samples shown as cluster tree (Figure 6) demonstrated that the three samples were divided into two clusters. S1 and S2 were clustered together, indicating a more similar fungi community structures and diversities. While as noncontaminated sample, S3 was divided into a separated group, implying distinguishing structures and diversities of fungi communities from the contaminated soils. Furthermore, the two clusters were well separated, which suggested a clear distinction of the fungi community structures and diversities between the two clusters [17].

In the present study, combined with the results of the high-throughput sequencing, further study should be carried out in the future to explore more microorganisms like bacteria and archaea with powerful features of oil resistance before strict screening, culturing, and domesticating of the dominant fungi genera.

## 4. Conclusions

This is the first study that evaluated the significant effects on physicochemical properties and fungal diversities of soil caused by AOS contamination. The results revealed that longtime oil exposure made the receiving soil arid, saline-alkali, and unsuitable for agriculture. The contents of both TPHs and HMs in the contaminated soils were apparently increased compared with noncontaminated soil. High-throughput sequencing results by Miseq platform showed significant changes in fungi community compositions and diversities. It was observed that oily circumstance could limit the growth of most genera and meanwhile promote the growth of certain oil-resistant fungi like *Venturia* (*Ascomycota*), *Alternaria* (*Ascomycota*), and *Piloderma* (*Basidiomycota*). In particular, there were three new-appeared genera in aged oil sludge-contaminated soils, among which *Cephalotheca* was identified as the core genus with the highest abundance of 16.76% in all the retained genera. The results could present a thorough understanding of microbial diversity in oil-contaminated soil and a better insight into the soil bioremediation.

## Figures and Tables

**Table 1:** Physicochemical properties of aged oil sludge-contaminated soil.

Sample	pH	Salinity (%)	MC^a^ (%)	TOC^b^ (%)	HMs^c^ (mg∙kg^−1^)			TPHs^d^ (mg∙kg^−1^)
					Cu	Zn	Cr	
S1	8.44	0.27	21.05	0.41	76.60	131.63	74.55	15.2
S2	8.56	0.36	22.55	0.35	47.93	93.81	111.46	13.6
S3	8.11	1.45	13.95	0.22	12.20	15.68	34.07	<5

^a^MC: moisture content; ^b^TOC: total organic carbon; ^c^HMs: heavy metals; ^d^TPHs: total petroleum hydrocarbons.

**Table 2:** Sequence information and fungal diversity indexes of samples.

Sample ID	Reads	0.97 (the similarity threshold of OTUs)					
		OTUs	Ace	Chao1	Coverage	Shannon-Weaver	Simpson
S1	31,118	475	476	478	0.999734	4.36	0.0467
S2	31,938	557	558	558	0.999812	4.61	0.0355
S3	33,275	565	567	566	0.999730	4.44	0.0414

**Table 3:** The fungal community structures and diversities at family level.

OTU ID	S1	S2	S3
Cephalothecaceae	16.76%	12.63%	0.00%
Aspergillaceae	10.76%	2.80%	11.32%
Unclassified	9.61%	7.63%	7.28%
Mortierellaceae	5.58%	12.74%	13.46%
Thelephoraceae	5.36%	2.18%	0.58%
Nectriaceae	4.22%	5.08%	5.24%
Cordycipitaceae	4.06%	3.09%	1.43%
Hypocreaceae	3.52%	1.11%	4.22%
Trichocomaceae	2.72%	2.36%	3.17%
Pleosporaceae	2.46%	5.44%	0.19%
Lasiosphaeriaceae	2.39%	6.26%	5.50%
Hypocreales_norank	2.27%	3.01%	3.85%
Pseudeurotiaceae	2.15%	0.91%	2.03%
Didymellaceae	1.91%	1.42%	0.80%
Chaetomiaceae	1.67%	2.97%	3.62%
Venturiaceae	1.60%	0.40%	0.08%
Stachybotriaceae	1.48%	0.33%	1.47%
Sporormiaceae	1.28%	0.35%	1.09%
Atheliaceae	1.21%	0.37%	0.10%
Sebacinaceae	1.17%	0.26%	0.16%
Botryosphaeriaceae	1.15%	3.09%	4.46%
Helotiales_norank	1.12%	0.92%	0.55%
Hyaloscyphaceae	0.86%	2.09%	2.01%
Cystofilobasidiaceae	0.82%	1.49%	1.38%
Herpotrichiellaceae	0.57%	0.59%	1.15%
Polyporales_norank	0.47%	1.04%	0.94%
Clavicipitaceae	0.42%	1.21%	0.72%
Phaeosphaeriaceae	0.34%	1.74%	0.06%
Meruliaceae	0.00%	0.00%	9.33%

## Data Availability

The data used to support the findings of this study are available from the corresponding author upon request.

## Abbreviations

ITS: International transcribed spacer
PHCs: Petroleum hydrocarbons
HMs: Heavy metals
AOS: Aged oil sludge
TPHs: Total petroleum hydrocarbons
OUT: Operational taxonomic unit
RDP: Ribosomal database project
TOC: Total organic carbon
PAHs: Polycyclic aromatic hydrocarbons
LME: Lignin-modifying enzymes
MC: Moisture content.
